# Highly Multiplexed Reverse-Transcription Loop-Mediated Isothermal Amplification and Nanopore Sequencing (LAMPore) for Wastewater-Based Surveillance

**DOI:** 10.1021/acsestwater.3c00690

**Published:** 2024-02-26

**Authors:** Seju Kang, Petra Choi, Ayella Maile-Moskowitz, Connor L. Brown, Raul A. Gonzalez, Amy Pruden, Peter J. Vikesland

**Affiliations:** † Department of Civil and Environmental Engineering, Virginia Tech, Blacksburg, Virginia 24061, United States; ‡ Virginia Tech Institute of Critical Technology and Applied Science (ICTAS), Sustainable Nanotechnology Center (VTSuN), Blacksburg, Virginia 24061, United States; § Department of Genetics, Bioinformatics, and Computational Biology, Blacksburg, Virginia 24061, United States; ∥ Hampton Roads Sanitation District, Virginia Beach ,Virginia23455, United States

**Keywords:** reverse-transcription loop-mediated isothermal amplification (RT-LAMP), nanopore sequencing, wastewater-based surveillance (WBS), SARS-CoV-2, multiplexing

## Abstract

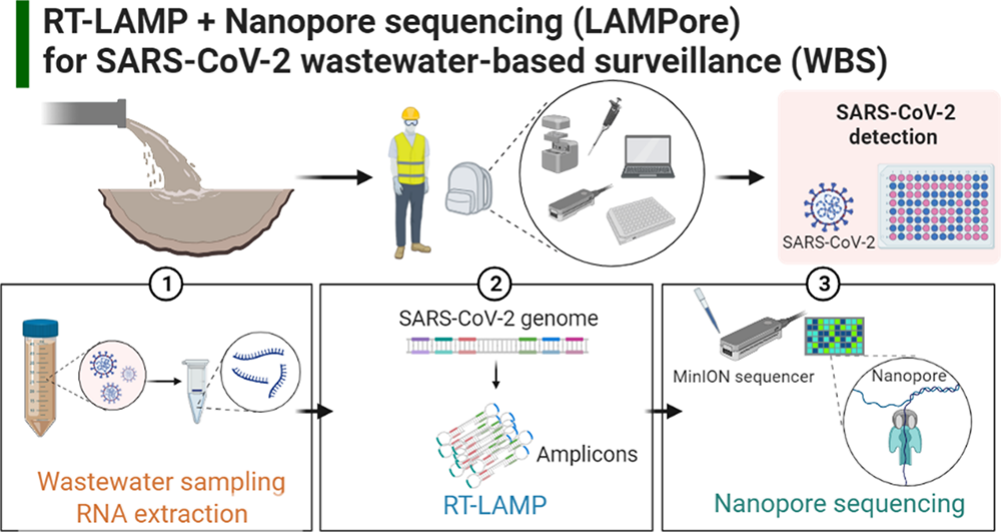

Wastewater-based surveillance (WBS) has gained attention as a strategy to monitor and provide an early warning for disease outbreaks. Here, we applied an isothermal gene amplification technique, reverse-transcription loop-mediated isothermal amplification (RT-LAMP), coupled with nanopore sequencing (LAMPore) as a means to detect SARS-CoV-2. Specifically, we combined barcoding using both an RT-LAMP primer and the nanopore rapid barcoding kit to achieve highly multiplexed detection of SARS-CoV-2 in wastewater. RT-LAMP targeting the SARS-CoV-2 N region was conducted on 96 reactions including wastewater RNA extracts and positive and no-target controls. The resulting amplicons were pooled and subjected to nanopore sequencing, followed by demultiplexing based on barcodes that differentiate the source of each SARS-CoV-2 N amplicon derived from the 96 RT-LAMP products. The criteria developed and applied to establish whether SARS-CoV-2 was detected by the LAMPore assay indicated high consistency with polymerase chain reaction-based detection of the SARS-CoV-2 N gene, with a sensitivity of 89% and a specificity of 83%. We further profiled sequence variations on the SARS-CoV-2 N amplicons, revealing a number of mutations on a sample collected after viral variants had emerged. The results demonstrate the potential of the LAMPore assay to facilitate WBS for SARS-CoV-2 and the emergence of viral variants in wastewater.

## Abstract 2

A new assay coupling RT-LAMP amplification and nanopore sequencing achieved multiplexed detection of SARS-CoV-2 and its sequence variations in wastewater.

## Introduction

Wastewater-based surveillance (WBS) is a promising tool to bolster community-level monitoring of infectious diseases.^1−3^ WBS is fundamentally achieved through detection of infectious disease biomarkers in sewage samples representative of a sewershed^4−7^ thus providing population-scale surveillance. WBS has shown promise as a strategy to inform interventions aimed at suppressing community disease transmission.^8,9^ For example, in response to the COVID-19 pandemic, WBS was rapidly implemented across the globe to detect SARS-CoV-2 viral RNA in wastewater and to provide an early warning of the spread of COVID-19 within a community.^1,10−13^ Ideally, WBS can be applied to relate loads of viral RNA in wastewater to fecal shedding of SARS-CoV-2 by infected members of the population. Encouragingly, in many cases, SARS-CoV-2 viral RNA loads in wastewater samples have been found to correlate with clinical COVID-19 cases in the community.^14^

Reverse-transcription polymerase chain reaction (RT-PCR)-based assays (e.g., RT-quantitative PCR, RT-digital droplet PCR) are now widely accepted as the gold standard approach for sensitive detection of SARS-CoV-2 viral RNA.^15−18^ Unfortunately, there were numerous PCR capacity shortages at the outset of large-scale testing (e.g., following the declaration of a global pandemic) since PCR requires centralized facilities and highly trained personnel. In particular, there have been analysis bottlenecks under limited resource conditions, especially those pervasive in low- and middle-income countries (LMICs).^19,20^ There has been rising demand for the development of rapid, alternative SARS-CoV-2 detection assays with low cost and high scalability. Moreover, the development of accessible point-of-use (POU) platforms has been demanded to achieve massive expansion of surveillance of SARS-CoV-2 and other infectious species.^8^ An additional challenge in molecular surveillance efforts has been the emergence of variants, which pose both additional public health risks and prompt tracking of mutations as a moving target for existing analytical assays.

Reverse-transcription loop-mediated isothermal amplification (RT-LAMP) has drawn attention as an alternative approach for SARS-CoV-2 detection.^21−25^ In a RT-LAMP assay, extracted RNA is first converted to complementary DNA (cDNA) through reverse transcription with a random primer and then amplified by LAMP. The LAMP assay that has come to be most typically used for SARS-CoV-2 detection relies upon six primers that recognize eight distinct regions of the SARS-CoV-2 genome. The targeted regions form a loop-shaped complex through primer hybridization and the strand displacement activity of *Bst* polymerase. The resultant loop complexes are replicated at constant temperature. The LAMP-based assay is technologically simpler and faster to perform than PCR and has higher amplification efficiency.^26,27^ Additionally, isothermal reaction conditions are more compatible for POU platforms because incubation does not require specialized instrumentation for precise temperature cycling, thus making the assay feasible for broad deployment.

Neither PCR- nor LAMP-based assays can directly distinguish sequence variants. However, next-generation sequencing (NGS) has been demonstrated to successfully detect and distinguish SARS-CoV-2 and its sequence variations in clinical and environmental samples.^28−32^ In particular, nanopore sequencing is attractive both because it yields long-read sequences and because it can be deployed in a portable hand-held format (i.e., Oxford Nanopore MinION).^33,34^ Nanopore sequencing uses flow cells that contain arrays of nanopores connected to electrodes that measure changes in electric current as nucleic acids flow through the nanopores. Characteristic raw electrical current signals are converted into a sequence of DNA bases. Recently, a SARS-CoV-2 detection assay was developed that coupled RT-LAMP with nanopore sequencing (LAMPore).^35−37^ The insertion of unique molecular barcodes (short specific nucleotide sequences) during cDNA amplification by RT-LAMP^38^ and library preparation for nanopore sequencing^39^ provides the capacity for highly multiplexed SARS-CoV-2 detection in clinical samples. However, the applicability of the LAMPore assay for SARS-CoV-2 detection in environmental samples that contain relatively low viral loads^40^ has not been explored and the potential to differentiate genomic heterogeneity, including single nucleotide polymorphisms (SNPs), has not yet been established.

In this study, we document for the first time the use of a LAMPore assay for detection of SARS-CoV-2 in wastewater. We anticipate that the highly multiplexed LAMPore assay will not only facilitate large-scale WBS for SARS-CoV-2, and other pathogens of interest, but also make tracking of emerging variants more accessible.

## Materials and Methods

**Figure 1:**
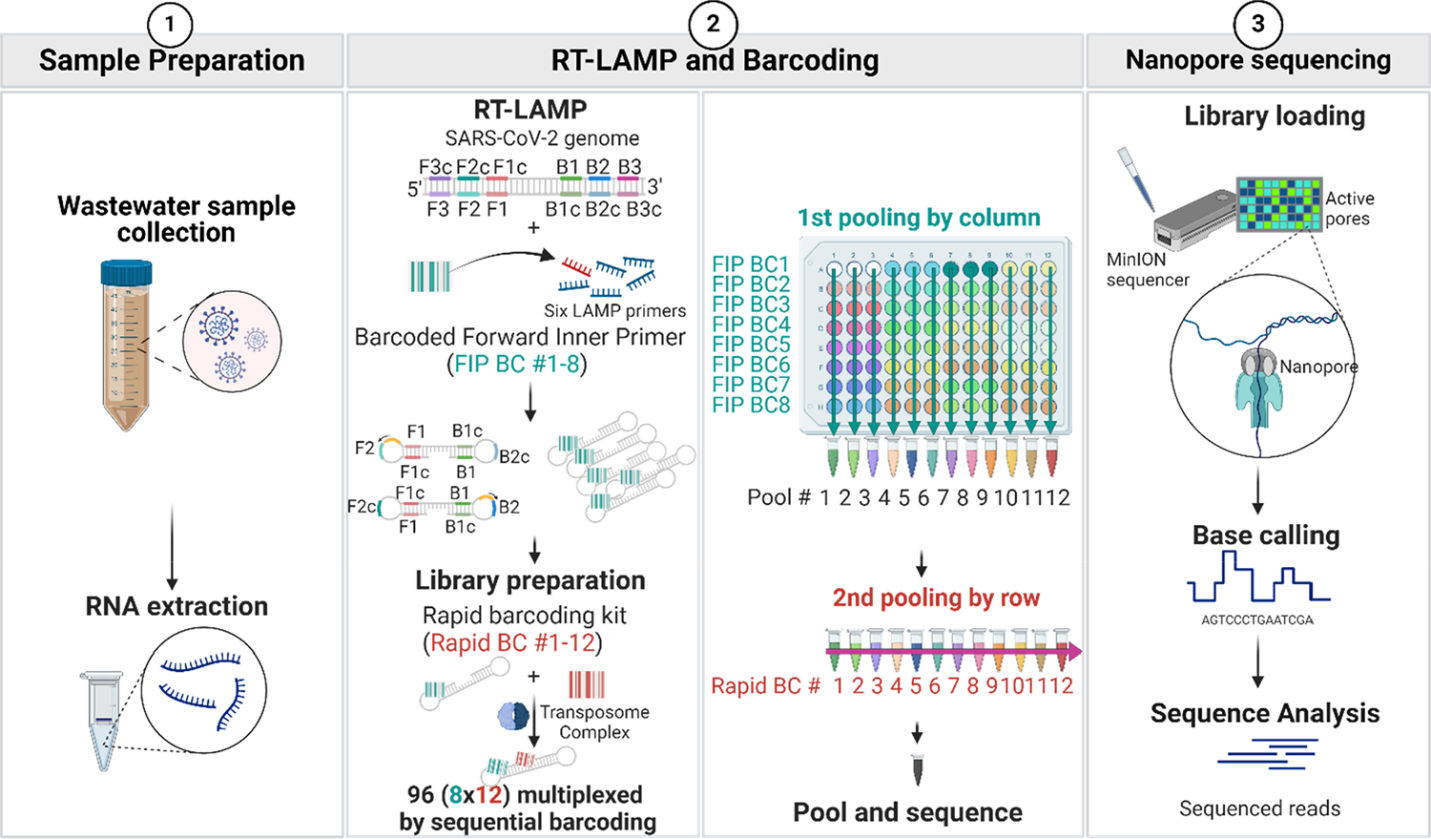
Workflow of the LAMPore assay for detection of SARS-CoV-2 in wastewater. (1) Wastewater sample collection and RNA extraction. (2) Sequential barcoding is conducted on 96 samples by using RT-LAMP forward inner primer (FIP) (8 barcodes in rows) and Rapid Barcoding Kit for nanopore sequencing (12 barcodes in columns) pooled together for analysis. (3) Library loading for nanopore sequencing and identification of SARS-CoV-2 reads by sequence analysis.

The workflow for the LAMPore assay is summarized in Figure 1 and detailed procedures for each step are summarized in the following sections.

### Wastewater Sample Collection and RNA Extraction

Between October and December 2020, 1 L grab sewage samples were collected once a week from a correctional facility experiencing an outbreak and were transported to the Virginia Tech (VT) laboratory on ice (Figure 1, step one). To detect SARS-CoV-2 variants, wastewater influent samples were collected between November and December 2022 from a manhole on the VT campus and at the inflow to the Christiansburg Wastewater Treatment Plant. Details on sample processing including MgCl_2_ addition, bovine coronavirus (BCoV) spiking, filtration, RNA extraction, and positive/negative controls are described in the Supporting Information.

### Reference RT-digital Droplet PCR (RT-ddPCR) Analysis

RT-ddPCR analysis was conducted on the wastewater RNA extracts to quantify the SARS-CoV-2 N gene.^41^ Details on the ddPCR analysis are described in the Supporting Information.

### RT-LAMP and Nanopore Sequencing

Ninety-six reactions were prepared for RT-LAMP in a 96-well plate (8 × 12; row × column). Two barcoding steps enabled 96 reactions to be pooled for analysis (Figure 1, step two): RT-LAMP (8 barcodes in rows) and Rapid Barcoding Kit (SQK-RBK004, Oxford Nanopore Technologies; ONT) for nanopore sequencing (12 barcodes in columns). Unique “barcode” sequences were embedded in the final products and can be sorted out from the sequenced reads. This approach enables pooling of multiple samples that can be sequenced together, thus reducing the cost per sample. Each 25 μL sample for RT-LAMP consisted of 12.5 μL of RT-LAMP Master Mix (E1700, New England Biolabs, Ipswich, MA), 2.5 μL of SARS-CoV-2 primer mix, 2.5 μL of BCoV primer mix, 7 μL of nuclease-free water, and 0.5 μL of RNA sample. Six primers (F3, B3, forward and backward inner primers (FIP and BIP), loop forward and backward (LF and LB) were designed to target the SARS-CoV-2 N region (accession NC_045512.2).^42^ The primer mix was prepared at 10× concentration (2 μM for F3 and B3; 16 μM for FIP and BIP; 8 μM for LF and LB). Separate primer mixes, each containing one of eight barcoded FIPs were used in the different rows. The primer sequences and eight barcodes are listed in Table S1 and the regions of the SARS-CoV-2 N gene targeted by the LAMP and PCR primers are highlighted in Figure S1.

The layout of the 96 RT-LAMP reactions in the 96-well plate for the first assay run on samples in 2020 is summarized in Figure S2. Briefly, we chose RNA extracts from 15 wastewater samples (WW #1–15) that were confirmed to be either SARS-CoV-2 positive or negative by the ddPCR assay (Table S2). RNA extracts from WW #1–10 were 10× diluted to minimize potential environmental inhibition of the RT-LAMP protocol, while RNA extracts from WW #11–15 were prepared at 5-, 10-, 20-, and 100× dilution to investigate how dilution impacts the LAMPore assay. Each RNA extract was run in triplicate to investigate the variability of the result across different barcoded products. Lastly, RNA extracts from heat-inactivated SARS-CoV-2 suspension and nuclease-free water were included as positive and negative controls, respectively.

The plate was incubated at 65 °C for 60 min. During this period, RNA was converted into cDNA and amplified by RT-LAMP. Following incubation, RT-LAMP products were pooled into 12 combined reaction mixtures by column. The concentrations in the pooled products were estimated by using a Qubit Fluorometer (ThermoFisher Scientific, Waltham, MA). An aliquot corresponding to 400 ng of DNA was taken from each of the 12 combined reaction mixtures. Barcoding was carried out following the manufacturer’s protocol for the Rapid Barcoding Kit, incorporating 12 barcoded adapters to the products during transpose-based fragmentation. Then, these 12 mixtures were pooled into one library that was loaded into the R9.4 flow cell (FLO-MIN106, ONT) of a MinION sequencer (Figure 1 - Step Three). Raw read data (FAST5 format) were submitted to the Sequence Read Archive (SRA) database at the National Center for Biotechnology Information (NCBI) (accession No. PRJNA875125).

### Sequence Analysis

Raw read data (FAST5 format) were base-called and trimmed into pass reads (FASTQ format) using the Guppy basecaller (ONT, v5.0.16). The barcodes added via (a) RT-LAMP primer and (b) Rapid Barcoding Kit for nanopore sequencing were demultiplexed to sort reads according to which of the 96 RT-LAMP products from which they originated. The 12 barcodes from Rapid Barcoding Kit were identified and classified using a Guppy barcoder (ONT, v5.0.16). To demultiplex the RT-LAMP barcodes, the alignment of the reads against the barcoded FIP sequences was conducted using vsearch (ver. 2.21.1.).^43^ Then, the SARS-CoV-2 positive reads for each sample were counted by aligning the reads against the corresponding regions of the SARS-CoV-2 genome targeted for RT-LAMP (i.e., amplicons) to reduce false-positive reads from nonspecific RT-LAMP amplification.^44−47^

### Statistical Analyses

The main criterion for scoring SARS-CoV-2 as detected was whether the number of reads annotated as SARS-CoV-2 in each sample was greater than a calculated read-count threshold. Because it is the more established assay and has a known highly sensitive detection limit, we assumed that ddPCR measurements reflect the true positive (TP) and negative (TN) measurements. The LAMPore assay false positives (FP) and negatives (FN) were accordingly scored against this assumption. Accordingly, the true-positive rate (TPR, sensitivity) and true-negative rate (TNR, specificity) of the LAMPore assay were calculated as TP/(TP + FN) and TN/(TN + FP), respectively. To optimize the read-count threshold, the F1 score and area under the curve (AUC) value were established.^35,48^ The F1 score is a metric calculated as follows: F1 score = 2 × TPR × TNR/ (TPR + TNR). The AUC value is another metric that indicates the area under the receiver operating characteristic curve and is obtained by plotting the TPR against the false negative rate, FNR = (1 – TNR). The optimal read-count threshold occurs when the F1 score and AUC value are maximized, thus, indicating the greatest accuracy of the assay.

### Profiling Sequence Variation among SARS-CoV-2 N Amplicons

To profile sequence variation among SARS-CoV-2 N amplicons, two compilations of the FASTQ base-called reads from wastewater samples collected during two separate LAMPore assay runs of samples collected in 2020 and 2022 were compared. The FASTQ base-called data only including the reads from wastewater samples were aligned against the SARS-CoV-2 genome and converted into SAM files using minimap2 (ver. 2.22-r1101).^49^ To provide visual alignment and assessment of the sequence variation among the reads, they were converted to BAM files using samtools (ver. 1.13)^50^ and uploaded to the Integrative Genomic Viewer (IGV; ver. 2.11.2) alignment software. Detailed information on the second assay run on the sample in 2022 is provided in the Supporting Information.

## Results and Discussion

### Validation and Optimization of the LAMPore Assay

To confirm amplification by RT-LAMP, and before barcoding for nanopore sequencing, we analyzed individual and pooled RT-LAMP products via gel electrophoresis (Figure S3). The gel exhibited ladder-like multiple bands at 100, 200, 300, 400, and 500 bps reflecting the various loop concatemers of RT-LAMP products that make it difficult to discriminate between specific and nonspecific LAMP amplification.^51^ This difficulty reflects the need to use approaches such as LAMPore to minimize the impacts of such nonspecific amplification.

Following validation of the RT-LAMP products, we conducted sequencing analysis of a series of alignments against (1) the barcodes incorporated via the Rapid Barcoding Kit, (2) the barcoded FIP sequences, and (3) the SARS-CoV-2 amplicon. This was done to sort each sample and identify them as one of the 96 products from which they originated and then count the corresponding number of SARS-CoV-2 reads. We optimized the alignment parameters (i.e., the length of the SARS-CoV-2 N amplicon and the alignment identity cutoffs) that generated the numbers of SARS-CoV-2 reads most closely correlating to the ddPCR results. The concentrations and their status (i.e., positive/negative) of SARS-CoV-2 and BCoV (spiked-in) in WW #1–15 as determined by ddPCR analysis are summarized in Tables S2 and S3. Among WW #1–15, five RNA extracts that had BCoV viral loads below the ddPCR detection limit were excluded since they reflected low viral recovery. Thus, 10 RNA extracts (*n* = 30, triplicate samples for each extract) were considered for optimization and evaluation. Details on optimization are provided in Tables S4–6. Finally, the potential inhibitive effects of the RNA extracts on the LAMPore assay were investigated. The LAMPore results for the samples at 5-, 10-, 20-, and 100-fold dilution were compared to the ddPCR results (Table S7). The results showed that 10- and 20-fold dilutions are optimal since they compared most favorably with the ddPCR assay results. This result suggests that some amount of dilution is required for RNA extracts from wastewater samples to prevent inhibition.

### Evaluation of the LAMPore Assay

**Figure 2:**
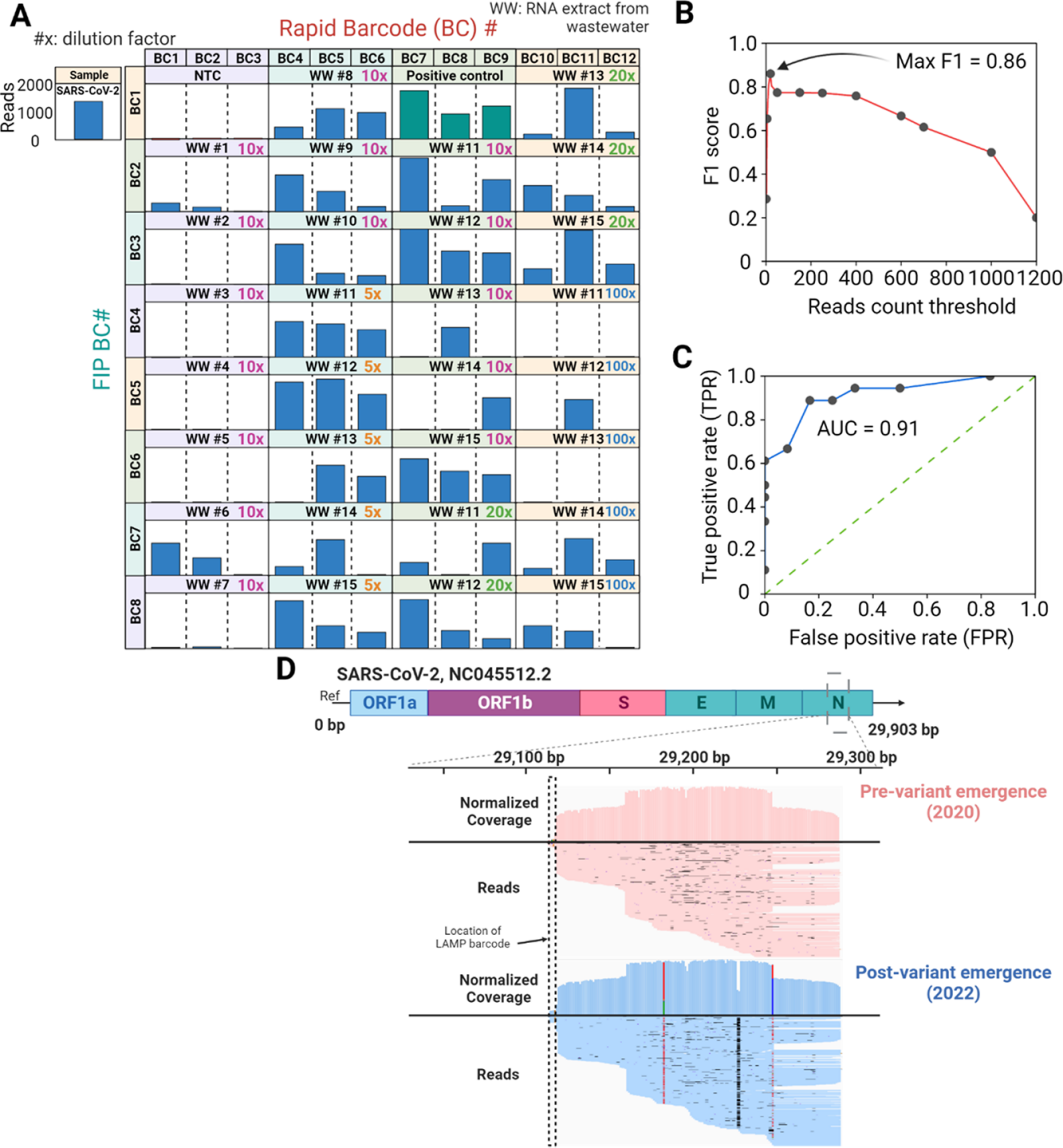
(A) Overall results of SARS-CoV-2 positive reads that correspond to 96 RT-LAMP products. (B and C) F1 score curve and receiver operating characteristic (ROC) curve with AUC value for the LAMPore assay. (D) IGV read alignment against SARS-CoV-2 reference genome for LAMPore assay on wastewater samples collected from pre- and postvariant emergence periods. Black lines indicate mismatched positions against the reference. Histogram on top of each compilation of aligned reads indicates normalized coverage of the reads with positions. Red (T), green (A), blue (C), and brown (G) colored bars indicate the base substitutions against the reference.

Figure 2A shows histograms of the SARS-CoV-2 reads across 96 RT-LAMP products under the optimized alignment parameters. As expected, negative controls were characterized by low numbers of reads (≤12), while positive controls had 1270 (±320, *n* = 3) reads. This result indicates that the concentration of SARS-CoV-2 in the samples is reflected by a greater number of reads. Overall, the number of reads for WW #1–15 at different dilution factors ranged from 0 to 2000. In some cases, we observed that the number of reads showed large coefficients of variability (∼169%) across triplicate samples. This may reflect barcode designs that are insufficiently different from each other^52^ as well as possible sequencing/base-calling errors.^53^

To establish criteria for scoring the presence/absence of SARS-CoV-2 in a sample, the number of reads from the LAMPore assay was dichotomized (i.e., digitalized) by setting a read-count threshold. We made decisions for WW #1–15 using the LAMPore assay and compared them with the results from the reference RT-ddPCR analysis. The F1 score and AUC value reveal the accuracy of the LAMPore assay at varying read-count thresholds (Figure 2B, C). Both the F1 score and AUC value were maximized at 0.86 and 0.91 with a read-count threshold of ∼20–50, indicating the greatest accuracy. The LAMPore assay results for the wastewater samples with a read-count threshold of 20 and the corresponding ddPCR results are summarized in Table 1. Of 30 samples, 26 were validated as true positives or negatives with a sensitivity of 89% and specificity of 83%.

**Table 1:** Comparison of the LAMPore versus the ddPCR Assays for SARS-CoV-2 Detection in the Wastewater Samples, with the Read-Count Threshold of 20

		LAMPore result		
		positive	negative	total
ddPCR result	positive	16	2	18
	negative	2	10	12
	total	18	12	30

### Detection of SNPs in SARS-CoV-2 N Amplicons

The alignment image indicates that the RT-LAMP products contained numerous copies of the SARS-CoV-2 N region with lengths of ∼100–200 bp in both forward and reverse orientations (Figure 2D). Transposase-based fragmentation by the Rapid Barcoding Kit resulted in fragments shorter than the bands in the gel. The amplified copies were situated within the region (29,119 to 29,287 bp) corresponding to the position targeted by the primers (Figure S1). The LAMP barcode was confirmed to be positioned before the targeted region began at 29,119 bp (Figure 2D). The multiple randomly distributed dots across the aligned reads indicate mismatches against the reference, likely due to sequencing error.

The histogram of each compilation of aligned reads indicates the normalized coverage at each genomic position. The average quality scores for the sequencing reads were 9.5 and 10.4 for samples in 2020 and 2022 (Figure S4). There are minimal consistent mutations within the wastewater samples collected in 2020, reflecting the fact that few known viral variants were circulating in the United States at that time. On the contrary, multiple predominant SNPs were found within wastewater samples collected in December 2022 at three positions: substitution at 29,182 (A > T, 69%); deletions at 29,226 and 29,227 (C and G, 54.8 and 56.7%); and substitution at 29,247 (C > T, 27%). These reflect samples that exceed the modest base-calling error rate (∼10%) of the ONT Nanopore platform with an R9.4 flow cell.^54^ This result demonstrates the capability of LAMPore assay-based profiling of targeted regions and ultimately variant monitoring in wastewater.

## Conclusions

WBS has drawn attention as a public health tool to monitor the community-level spread of SARS-CoV-2 and more recently influenza and respiratory syncytial virus.^7,55^ Here, we have demonstrated that a multiplexed LAMPore assay can successfully detect and differentiate SARS-CoV-2 variants in wastewater samples, thus illustrating its promise as a tool for WBS. Sequencing SARS-CoV-2 genes using the LAMPore assay enabled the robust monitoring of base mutations. We suggest that the LAMPore assay can facilitate large-scale WBS considering the high capacity for multiplexing and potential for prompt variant tracking at community scale (Time to result: 2.5 h = 1 h of RT-LAMP + 15 min for library preparation +1 h of Nanopore sequencing). The cost for the LAMPore assay is estimated to be ∼$12.00 per sample (∼$4.50/sample for extraction; $0.73/sample for reagents; $6.75/sample for sequencing), with the assumption of one-time flow cell use. It is expected that additional multiplexing and reuse of the flow cell can further reduce the assay cost. To make WBS more accessible, particularly when used to monitor emerging variants of SARS-CoV-2 or other infectious disease agents, the application of multiple targeted LAMPore assays will be required.

Despite the success of LAMPore for wastewater samples, some issues require further efforts to improve the assay. For example, to improve the scalability of the LAMPore assay and to reduce sample analysis costs, it is important to develop a barcode design system that can minimize “barcode” leakage due to sequencing errors.^52^ The relatively large variabilities observed across replicate samples with different barcodes observed herein require further attention. Similarly, standardized sequence analysis (e.g., alignment parameters) are desirable to increase reproducibility, accessibility, throughput, and time to result.

## Supporting Information Available

(1) Experimental details on sample processing; (2) reference ddPCR analysis; (3) RT-LAMP primer sequences for detection of SARS-CoV-2 and bovine coronavirus (BCoV) and 10-nt barcodes in FIP; (4) LAMP and PCR primer alignments against SARS-CoV-2 N region; (5) layout of 96-well plate that contains 96 RT-LAMP assays; (6) additional LAMPore run on December 2022 wastewater samples; (7) gel electrophoresis of the 12 pooled samples from 96 RT-LAMP products; (8) RT-ddPCR analysis results targeting the SARS-CoV-2 N region for the wastewater samples; (9) RT-ddPCR analysis results targeting bovine coronavirus (BCoV) N region for the wastewater samples; (10) alignment parameter optimization; (11) SARS-CoV-2 positive reads with alignment against the short, medium, and long amplicons (identity ≥0.8); (12) SARS-CoV-2 positive reads with alignment against barcoded FIP sequences with different identities; (13) SARS-CoV-2 positive reads with alignment against short amplicon with different identities; (14) SARS-CoV-2 positive reads at 5-, 10-, 20-, and 100-fold dilutions; and (15) quality score distribution over all sequences and the boxplots of quality scores across all bases for the sequencing reads (PDF)
