# Supplementary material for: Highly Multiplexed Reverse-Transcription Loop-Mediated Isothermal Amplification and Nanopore Sequencing (LAMPore) for Wastewater-Based Surveillance
Source: ACS ES T Water. 2024 Feb 26;4(4):1629–36. doi: 10.1021/acsestwater.3c00690 (PMC11019537; doi:10.1021/acsestwater.3c00690)
Supplement: Supplementary file 1 — ew3c00690_si_001.pdf [file ew3c00690_si_001.pdf]

Supporting Information

Highly Multiplexed Reverse-TranscriptionL-mediated Isothermal Amplification and Nanopore Sequencing (LAMPore) for SARS-CoV-2 Wastewater-Based Surveillance

Seju Kang[a,b], Petra Choi[a,b], Ayella Maile-Moskowitz[a,b], Connor L. Brown[c], , Raul A. Gonzalez[d], Amy Pruden[a,b], Peter J. Vikesland[a,b*]

[a]Department of Civil and Environmental Engineering, Virginia Tech, Blacksburg, Virginia 24061, United States;

[b]Virginia Tech Institute of Critical Technology and Applied Science (ICTAS) Sustainable Nanotechnology Center (VTSuN), Blacksburg, Virginia 24061, United States;

[c]Department of Genetics, Bioinformatics, and Computational Biology, Blacksburg, Virginia 24061, United States;

[d]Hampton Roads Sanitation District, Virginia Beach, Virginia 23455, United States

[*] Corresponding author: pvikes@vt.edu

Postal address: 415 Durham, Virginia Tech, Blacksburg 24061, VA, USA

Keywords: *Reverse-transcription loop-mediated isothermal amplification (RT-LAMP), nanopore sequencing, wastewater-based surveillance, SARS-CoV-2, multiplexing*

Total Pages: 18

**Experimental description on sample processing**	**Page S2**
**Reference ddPCR analysis**	**Page S3**
**Tables S1**	**Page S4**
**Figure S1**	**Page S5**
**Figure S2**	**Page S6**
**LAMPore assay on December 2022 wastewater samples**	**Page S7**
**Figure S3**	**Page S8**
**Tables S2-S3**	**Pages S9-S10**
**Alignment parameter optimization**	**Page S11**
**Tables S4-S8**	**Pages S12-S16**
**Figure S4**	**Page S17**
**SI References**	**Page S18**

Experimental description of sample processing

Each sample was processed via electronegative filtration as previously described.[1,2] In brief, $MgCl_2$ was added to 150−200 mL of sample to a final concentration of 25 mM and the pH was adjusted to 3−4 to facilitate attachment of virus particles to biomass via salt-bridging.[3] An aliquot of 1 μL/mL of calf-guard cattle vaccine (Zoetis, Parsippany-Troy Hills, NJ) was spiked into each sample as a bovine coronavirus (BCoV) source to validate filtration and extraction recovery efficiency. Samples were filtered through 0.45-μm mixed cellulose ester membrane filters (Millipore, Billerica, MA) and the filters were folded and torn into ~1 cm^2 pieces using sterile forceps. RNA extraction was conducted following a TRIzol/ethanol-based nucleic acid extraction protocol in a 96-well format system (Zymo Research) as described previously.[2] As a positive control, RNA was extracted from heat-inactivated SARS-CoV-2 suspension (VR1986HK, ATCC) using a QIAamp mini viral RNA kit (Cat. No. 52904, Qiagen, Hilden, Germany). Negative controls were prepared using nuclease-free water (Invitrogen, Waltham, MA).

Reference RT-ddPCR analysis

For ddPCR analysis, subsets of RNA extracts were shipped on dry ice to Hampton Roads Sanitation District (HRSD), Virginia Beach, VA. Details on ddPCR analysis using the Bio-Rad QX200 (Bio-Rad, Hercules, CA, USA) are provided elsewhere.[2] Simply, per sample, the 20-µL final reaction volume for RT-ddPCR consisted of 5 µL of One-step RT-ddPCR Supermix, 2 µL of reverse transcriptase, 1 µL of 300 mM dithiothreitol (DTT), 3 µL of primer mix (final concentration: 900 nM forward/reverse primer each and 250 nM probe), 5 µL of nuclease-free water, and 4 µL of RNA extract. The sequences of primers and probe are provided in previous study.[2] ddPCR targeting the SARS-CoV-2 N region was conducted. The concentration of SARS-CoV-2 N genes (viral copies/100 mL) were quantified based on the number of droplets showing positive signals out of ≥10,000 total droplets. BCoV N genes were quantified to validate filtration and extraction recovery rates.

The samples were considered positive when the concentration was >2× the limit of detection (LOD). LODs were calculated by running serial dilutions of Twist Synthetic SARS-CoV-2 RNA Control 4 (Twist Bioscience, San Francisco, CA) in seven replicates over six orders of magnitude. The LOD was the concentration at which > 60% of the replicates were positive.

The concentration of positive RNA control that was extracted from heat-inactivated SARS-CoV-2 suspension was estimated to be 511.35 gc/µL. The concentrations of SARS-CoV-2 in WW #1-15 and their status (i.e., positive/negative) as determined by the reference ddPCR analysis are summarized in **Table S2.**

Table S1. RT-LAMP primer sequences for detection of SARS-CoV-2 and bovine coronavirus (BCoV) and 10-nt barcodes in FIP

Target gene	Primer	Sequences (5'-3')	Length (bp)	Ref.
SARS-CoV-2 N	F3	AACACAAGCTTTCGGCAG	18	4
	B3	GAAATTTGGATCTTTGTCATCC	22	
	FIP	TGCGGCCAATGTTTGTAATCAG **XXXXXXXXXX** CCAAGGAAATTTTGGGGAC	51	
	BIP	CGCATTGGCATGGAAGTCACTTTGATGGCACCTGTGTAG	39	
	LF	TTCCTTGTCTGATTAGTTC	19	
	LB	ACCTTCGGGAACGTGGTT	18	
Bovine coronavirus (BCoV) N	F3	AGCGTCAATTGCTACCACG	19	Designed in this study
	B3	AGACCTTCCTGAGCCTTCAA	20	
	FIP	GCGACCCAGTAGACTCCGTCAA **XXXXXXXXXX** CTATCTTGGAACAGGACCGC	52	
	BIP	CTGACATTCTCGATCGGGACCCACCCTGAGGGAGTACCGT	40	
**Barcodes in FIP**				
Barcode 1		**GTGGCAATAA**	10	5
Barcode 2		**CTACGCAAGC**		
Barcode 3		**GCTCGAAGAA**		
Barcode 4		**GATACACACT**		
Barcode 5		**AGGAGACGG**A		
Barcode 6		**CTCCCGTTTA**		
Barcode 7		**CTGTGAGATC**		
Barcode 8		**ACCCAGAAGC**		

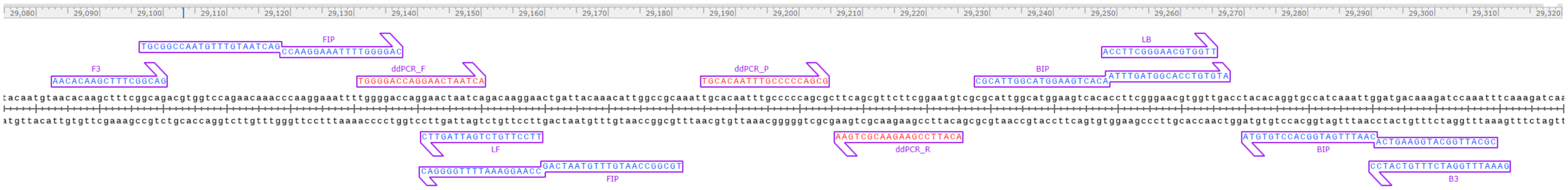

Figure S1. Alignments for RT-LAMP and RT-PCR primers (blue and red colored) against SARS-CoV-2 N gene.

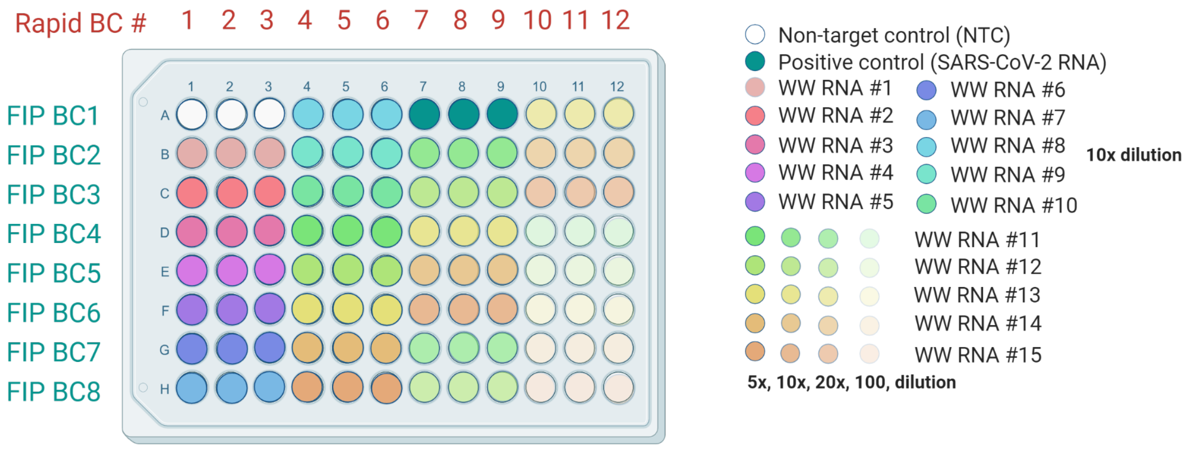

Figure S2. Layout of 96-well plate that contains 96 RT-LAMP assays. Barcodes (BCs) from FIP for RT-LAMP and Rapid Barcoding Kit for nanopore sequencing were added to the RT-LAMP product before sequencing.

Additional LAMPore assay on December 2022 wastewater samples

To obtain SARS-CoV-2 positive reads after the variant emergence period, additional wastewater samples were collected in December 2022. Three additional wastewater from the manhole at VT laboratory for three consecutive days (November 30th, December 1st and 2nd; WW RNA #1-3) and one from Christiansburg Wastewater Treatment Plant (WW RNA #4) on November 30th were grab-sampled and transported to the laboratory on ice. The RNA extraction, RT-LAMP, and Nanopore sequencing analysis were conducted following the same procedure. The layout of 96 RT-LAMP assays is shown below including four wastewater RNA extracts with multiple dilutions, non-target control (NTC), positive control, BCoV-spiked control, and extraction control in replicates. The concentrations of WW RNA #1-4 were measured to be 4.14, 3.63, 2.42, and 6.38 $\times 10^2$ viral copies in 100 mL wastewater using ddPCR analysis.

For comparison of wastewater samples for pre- and post-variant emergence period, the reads from Columns 4-6 and 10-12 in **Figure S2** and from Columns 9-12 in the below Figure were visually aligned against the SARS-CoV-2 reference genome using the Integrative Genomic Viewer (IGV; ver. 2.11.2) alignment software.

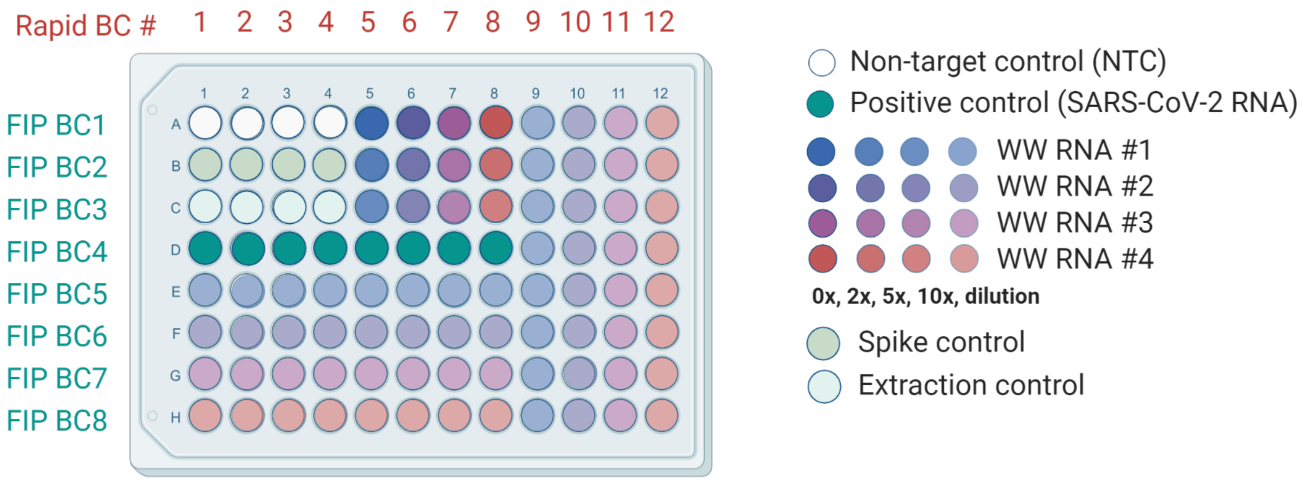

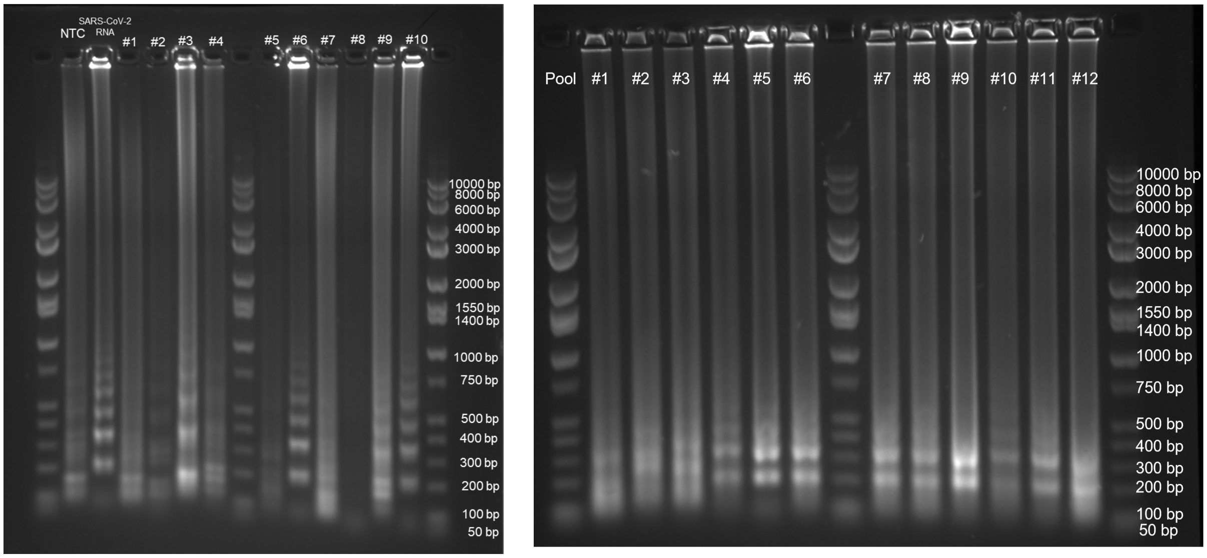

Figure S3 Gel electrophoresis of the individual (left) and pooled (right) RT-LAMP products

Gel electrophoresis was run for 10 of individual (left) and 12 of pooled RT-LAMP products by columns (right) to validate sample amplification by the RT-LAMP procedure. Multi-ladder-like bands with strong intensity were observed, thus demonstrating successful amplification by RT-LAMP.[6] Unlike PCR, gel electrophoresis of RT-LAMP products is challenging since the various loop concatemers make it difficult to discriminate between specific and non-specific amplification.[7]

Table S2. RT-ddPCR analysis results targeting the SARS-CoV-2 N region for the wastewater samples

	Viral copies in 100 mL	Limit of Detection (LOD)	Status
WW #1	2.72×10^{2}	2.67×10^{2}	Negative
WW #2	0	1.38×10^{2}	Negative
WW #3	4.16×10	8.02×10	Negative
WW #4	4.24×10	4.09×10	Negative
WW #5	1.60×10^{3}	6.92×10	Positive
WW #6	5.02×10	2.30×10	Positive
WW #7	4.29×10^{5}	1.01×10^{3}	Positive
WW #8	2.02×10^{4}	1.08×10^{2}	Positive
WW #9	2.00×10^{2}	1.08×10^{2}	Positive
WW #10	8.18×10^{2}	1.05×10^{2}	Positive
WW #11	5.09×10^{3}	1.07×10^{2}	Positive
WW #12	7.03×10^{3}	1.07×10^{2}	Positive
WW #13	3.99×10^{3}	7.11×10	Positive
WW #14	7.53×10^{3}	7.11×10	Positive
WW #15	3.92×10^{5}	9.63×10	Positive

Table S3. RT-ddPCR analysis results targeting bovine coronavirus (BCoV) N region for the wastewater samples

	Viral copies in 100 mL	
WW #1	4.01×10^5	
WW #2	2.96×10^2	
WW #3	3.80×10^4	
WW #4	9.15×10^4	
WW #5	4.1×10	Below the detection limit
WW #6	1.46×10^3	
WW #7	0	Below the detection limit
WW #8	4.20×10^5	
WW #9	2.25×10^5	
WW #10	3.71×10^5	
WW #11	1.30×10	Below the detection limit
WW #12	2.17×10	Below the detection limit
WW #13	3.15×10^5	
WW #14	1.40×10^4	
WW #15	0	Below the detection limit

Alignment parameter optimization

We optimized the alignment parameters (i.e., the length of the SARS-CoV-2 amplicon and the alignment identity cut-offs) that generated the numbers of SARS-CoV-2 reads most closely correlating to the ddPCR results of detection. Samples that had BCoV viral loads below the ddPCR detection limit (**Table S3**) were excluded from the LAMPore assay decision tree since they reflected low viral recovery. The SARS-CoV-2 amplicon developed via primer design spans F2, F1, B1c, B2c, and B1 (**Figure 1A**- Step Two). First, we aligned the reads against three different length amplicons: 188 bp-long (F2 to B1), 129 bp-medium (F2 to B1c), and 88 bp-short (F1 to B1c) amplicons (**Table S4**). Amplicon length had no significant effect on the numbers of counted reads across the samples. Second, the alignment identities for the barcoded FIP sequences and SARS-CoV-2 amplicon were optimized (**Table S5** and **S6**).

Table S4. SARS-CoV-2 positive reads with alignment against the short, medium, and long amplicons (identity ≥ 0.8)

Amplicon	Positive reads		
**Long**	**1**	**2**	**3**
WW #1	0	0	2
WW #2	0	1	1
WW #3	1	2	0
WW #4	0	2	0
WW #6	247	155	1
WW #8	0	246	208
WW #9	224	0	1
WW #10	319	1	0
WW #13	0	239	0
WW #14	0	0	278
**Medium**			
WW #1	0	1	2
WW #2	0	1	1
WW #3	1	1	0
WW #4	1	0	0
WW #6	263	162	1
WW #8	0	261	227
WW #9	241	0	1
WW #10	346	2	0
WW #13	0	255	0
WW #14	0	0	294
**Short amplicon**			
WW #1	0	1	2
WW #2	0	1	1
WW #3	1	1	0
WW #4	1	2	0
WW #6	323	188	1
WW #8	0	295	262
WW #9	274	1	1
WW #10	386	2	0
WW #13	0	280	0
WW #14	0	0	333

With the read-count threshold of 20, 20 out of 30 samples returned true positives/negatives against short, medium, and long amplicons. (Identity cutoff = 0.8 for amplicon and identity cutoff = 1.0 for barcoded-FIP sequences). There was no significant difference across different length of amplicons. Thus, short amplicon was chosen for the following parameter optimization.

Table S5. SARS-CoV-2 positive reads with alignment against barcoded-FIP sequences with different identities

Identity	Positive reads		
**1.0**	**1**	**2**	**3**
WW #1	0	1	2
WW #2	0	1	1
WW #3	1	1	0
WW #4	1	2	0
WW #6	323	188	1
WW #8	0	295	262
WW #9	274	1	1
WW #10	386	2	0
WW #13	0	280	0
WW #14	0	0	333
**>0.9**			
WW #1	346	211	7
WW #2	12	13	5
WW #3	5	3	0
WW #4	6	4	0
WW #6	1143	630	3
WW #8	468	1088	950
WW #9	1355	705	200
WW #10	1424	401	350
WW #13	22	1078	29
WW #14	21	12	1154

With the read-count threshold of 20, 20 and 26 out of 30 samples returned true positives/negatives with the identity cutoff of 1.0 and 0.9 for barcoded-FIP sequences. The identity cutoff of 0.9 for barcoded-FIP sequences was chosen since it showed greater number of samples with true positives/negatives.

Table S6. SARS-CoV-2 positive reads with alignment against short amplicon with different identities

Identity	Positive reads		
**≥0.8**	**1**	**2**	**3**
WW #1	346	211	7
WW #2	12	13	5
WW #3	5	3	0
WW #4	6	4	0
WW #6	1143	630	3
WW #8	468	1088	950
WW #9	1355	705	200
WW #10	1424	401	350
WW #13	22	1078	29
WW #14	21	12	1154
**≥0.7**			
WW #1	355	220	7
WW #2	12	13	6
WW #3	5	3	0
WW #4	7	4	0
WW #6	1179	658	3
WW #8	473	1120	978
WW #9	1389	732	210
WW #10	1458	411	359
WW #13	22	1108	29
WW #14	21	12	1184
**≥0.6**			
WW #1	363	225	17
WW #2	12	61	159
WW #3	5	260	6
WW #4	7	7	13
WW #6	1189	663	4
WW #8	473	1129	980
WW #9	1735	739	237

WW #10	1463	413	360
WW #13	22	1127	29
WW #14	21	13	1191
**≥0.5**			
WW #1	419	255	80
WW #2	15	968	887
WW #3	5	593	15
WW #4	7	14	683
WW #6	1197	676	6
WW #8	473	1134	985
WW #9	2140	741	956
WW #10	1465	415	361
WW #13	29	1134	30
WW #14	22	13	1197

With the read-count threshold of 20, 26 out of 30 samples returned true positives/negatives with the identity cutoff of 0.8 and 0.7 for amplicon. And 23 and 21 out of 30 samples returned true positives/negatives with the identity cutoff of 0.7 and 0.6. The identity cutoff of 0.8 for amplicon was chosen since it showed greater number of samples with true positives/negatives.

Table S7. SARS-CoV-2 positive reads at 5-, 10-, 20-, and 100-fold dilutions of RNA extracts from wastewater samples

Dilution	Positive reads		
**5-fold**	**1**	**2**	**3**
WW RNA #13	9	1368	962
WW RNA #14	312	1287	7
**10-fold**			
WW RNA #13	22	1078	29
WW RNA #14	21	12	1954
**20-fold**			
WW RNA #13	213	1800	237
WW RNA #14	943	579	194
**100-fold**			
WW RNA #13	3	9	3
WW RNA #14	270	1326	561

With the read-count threshold of 20, 5 and 6 out of 6 samples returned true positives at 10- and 20-fold dilution. But, only 4 and 3 out 6 samples returned true positives at 5- and 100-fold dilutions.

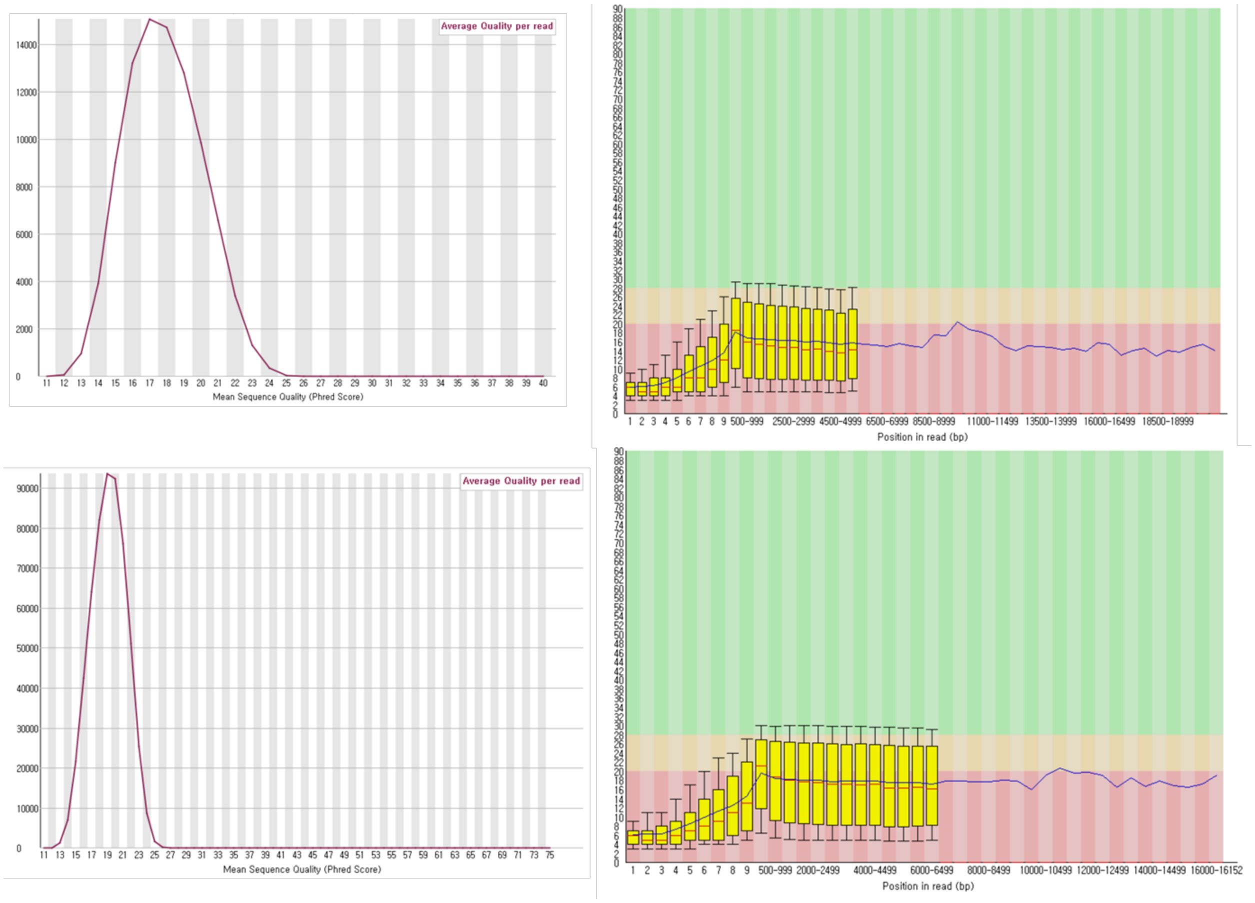

Figure S4 Quality score distribution over all sequences (left) and the boxplots of quality scores across all bases (right) for the sequencing reads from the samples in 2020 (top) and 2022 (bottom).

References

1 W. Ahmed, P. M. Bertsch, A. Bivins, K. Bibby, K. Farkas, A. Gathercole, E. Haramoto, P. Gyawali, A. Korajkic, B. R. McMinn, J. F. Mueller, S. L. Simpson, W. J. M. Smith, E. M. Symonds, K. V. Thomas, R. Verhagen and M. Kitajima, *Sci. Total Environ.*, 2020, **739**, 139960.

2 A. Cohen, A. Maile-Moskowitz, C. Grubb, R. A. Gonzalez, A. Ceci, A. Darling, L. Hungerford, R. D. Fricker, C. V. Finkielstein, A. Pruden and P. J. Vikesland, *ACS ES&T Water*, , DOI:10.1021/acsestwater.2c00059.

3 E. Haramoto, B. Malla, O. Thakali and M. Kitajima, *Sci. Total Environ.*, 2020, **737**, 140405.

4 J. P. Broughton, X. Deng, G. Yu, C. L. Fasching, V. Servellita, J. Singh, X. Miao, J. A. Streithorst, A. Granados, A. Sotomayor-Gonzalez, K. Zorn, A. Gopez, E. Hsu, W. Gu, S. Miller, C.-Y. Pan, H. Guevara, D. A. Wadford, J. S. Chen and C. Y. Chiu, *Nat. Biotechnol.*, 2020, **38**, 870–874.

5 K. U. Ludwig, R. M. Schmithausen, D. Li, M. L. Jacobs, R. Hollstein, K. Blumenstock, J. Liebing, M. Słabicki, A. Ben-Shmuel, O. Israeli, S. Weiss, T. S. Ebert, N. Paran, W. Rüdiger, G. Wilbring, D. Feldman, B. Lippke, N. Ishorst, L. M. Hochfeld, E. C. Beins, I. H. Kaltheuner, M. Schmitz, A. Wöhler, M. Döhla, E. Sib, M. Jentzsch, J. D. Borrajo, J. Strecker, J. Reinhardt, B. Cleary, M. Geyer, M. Hölzel, R. Macrae, M. M. Nöthen, P. Hoffmann, M. Exner, A. Regev, F. Zhang and J. L. Schmid-Burgk, *Nat. Biotechnol.*, 2021, **39**, 1556–1562.

6 P. C. Foo, A. B. Nurul Najian, N. A. Muhamad, M. Ahamad, M. Mohamed, C. Yean Yean and B. H. Lim, *BMC Biotechnol.*, 2020, **20**, 34.

7 J. Wadden, B. S. Newell, J. Bugbee, V. John, A. K. Bruzek, R. P. Dickson, C. Koschmann, D. Blaauw, S. Narayanasamy and R. Das, *Commun. Biol.*, 2022, **5**, 708.
